# 8-Methyl-5-methyl­ene-2-oxotricyclo[5.3.1.1^3,9^]dodecan-*endo*-8-ol

**DOI:** 10.1107/S1600536808009677

**Published:** 2008-04-16

**Authors:** Isa Y. H. Chan, Roger Bishop, Donald C. Craig, Marcia L. Scudder, Weimin Yue

**Affiliations:** a School of Chemistry, University of New South Wales, Sydney 2052, Australia; b School of Pharmacy, East China University of Science and Technology, 130 Meilong Road, Shanghai 200237, People’s Republic of China

## Abstract

The title compound, C_14_H_20_O_2_, crystallizes with homochiral chains of mol­ecules hydrogen bonded together along the *b* axis. Adjacent chains in the *ab* plane contain mol­ecules of the same chirality, leading to a chiral segregation of the mol­ecules into layers.

## Related literature

For related literature, see: Yue *et al.* (2002 ▶, 2006 ▶, 2007 ▶, 1997 ▶, 2000 ▶).
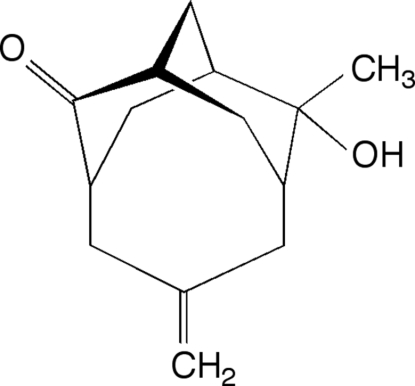


## Experimental

### 

#### Crystal data

C_14_H_20_O_2_
*M* *_r_* = 220.3
Monoclinic, 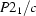

*a* = 7.554 (3) Å
*b* = 13.196 (3) Å
*c* = 12.597 (5) Å
β = 108.16 (2)°
*V* = 1193.2 (7) Å^3^
*Z* = 4
Mo *K*α radiation
μ = 0.08 mm^−1^
*T* = 294 K
0.25 × 0.20 × 0.20 mm

#### Data collection

Enraf–Nonius CAD-4 diffractometer
Absorption correction: none
2247 measured reflections
2079 independent reflections
1296 reflections with *I* > 2σ(*I*)
*R*_int_ = 0.016
1 standard reflection frequency: 30 min intensity decay: none

#### Refinement

*R*[*F* ^2^ > 2σ(*F* ^2^)] = 0.049
*wR*(*F* ^2^) = 0.052
*S* = 1.27
2079 reflections
145 parameters
H-atom parameters constrained
Δρ_max_ = 0.28 e Å^−3^
Δρ_min_ = −0.28 e Å^−3^

### 

Data collection: *CAD-4 Software* (Enraf–Nonius, 1989 ▶); cell refinement: *CAD-4 Software*; data reduction: local program; program(s) used to solve structure: *SIR92* (Altomare *et al.*, 1994 ▶); program(s) used to refine structure: *RAELS* (Rae, 2000 ▶); molecular graphics: *ORTEPII* (Johnson, 1976 ▶) and *CrystalMaker* (Palmer, 2005 ▶); software used to prepare material for publication: local programs.

## Supplementary Material

Crystal structure: contains datablocks global, I. DOI: 10.1107/S1600536808009677/hg2391sup1.cif

Structure factors: contains datablocks I. DOI: 10.1107/S1600536808009677/hg2391Isup2.hkl

Additional supplementary materials:  crystallographic information; 3D view; checkCIF report

## Figures and Tables

**Table 1:** Hydrogen-bond geometry (Å, °)

*D*—H⋯*A*	*D*—H	H⋯*A*	*D*⋯*A*	*D*—H⋯*A*
O1—H1O1⋯O2^i^	1.00	1.87	2.867 (4)	180

Symmetry code: (i) 
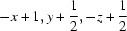
.

**Fig. 1.**
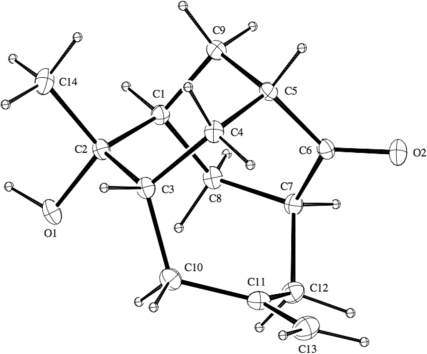
Molecular structure of the compound, with ellipsoids drawn at 30% probability level.

**Fig. 2.**
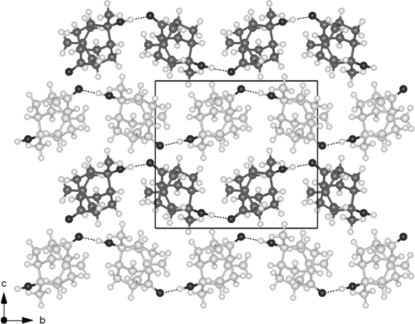
Unit cell diagram showing the O—H···O═C hydrogen bonded chain along b. Adjacent layers along c are chirally pure with alternating chirality. This is indicated by light and dark shading of C atoms.

## supplementary crystallographic information

### Comment

The preparation of this compound is part of a project involving alicyclic diols (Yue *et al.* 2002, 2006, 2007). The title compound (Fig. 1) crystallizes in space group *P*2_1_/*c* with homochiral chains of molecules along *b* which are held together by O—H···O═C hydrogen bonding. Adjacent chains along *a* are of the same chirality, leading to chirally pure layers within the crystal which are shaded light and dark in Fig. 2. It is unusual to observe chirally pure layers within a centrosymmetric lattice. In this case the layer is generated by a combination of the 2_1_ screw axis along *b* and translation along the short *a* axis, neither of which generates a change in chirality.

### Experimental

5-Methylidenetricyclo[5.3.1.1^3,9^]dodecane-2,8-dione (Yue *et al.*, 1997, 2000) was reacted with *ca* 1 equivalent of methyllithium in tetrahydrofuran solution. After standard work up of the reaction, the crude solid product was recrystallized to afford the title compound of m.p. 105–107°C. ^13^C NMR (75.5 MHz, CDCl_3_) δ: 28.1 (CH_2_), 30.9 (CH_2_), 31.9 (CH_3_), 34.7 (CH_2_), 38.6 (CH_2_), 38.8 (CH), 40.6 (CH), 42.7 (CH), 43.3 (CH), 45.3 (CH_2_), 74.8 (C), 119.3 (CH_2_), 148.2 (C), 219.9 (C). ^1^H NMR (300 MHz, CDCl_3_) δ: 1.44 (s, 3H), 1.83–1.98 (m, 4H), 2.01–2.21 (m, 4H), 2.29–2.47 (m, 4H), 2.66–2.76 (m, 2H), 2.99 (dd, *J* = 14.3, 7.1 Hz, 1H), 4.94 (d, *J* = 15.1 Hz, 2H). X-ray quality crystals were obtained from diethyl ether solution.

### Refinement

Hydrogen atoms attached to C were included at calculated positions (C—H = 1.0 Å). The hydroxy hydrogen atom was located on a difference map, and was then fixed at a position along the O···O vector with O—H = 1.0 Å. All hydrogen atoms were refined with isotropic thermal parameters equivalent to those of the atom to which they were bonded.

### Crystal data

**Table:**

C_14_H_20_O_2_	*F*_000_ = 480.0
*M**_r_* = 220.3	*D*_x_ = 1.23 Mg m^−^^3^
Monoclinic, *P*2_1_/*c*	Mo *K*α radiation λ = 0.71073 Å
*a* = 7.554 (3) Å	Cell parameters from 11 reflections
*b* = 13.196 (3) Å	θ = 10–11º
*c* = 12.597 (5) Å	µ = 0.08 mm^−^^1^
β = 108.16 (2)º	*T* = 294 K
*V* = 1193.2 (7) Å^3^	Block, colourless
*Z* = 4	0.25 × 0.20 × 0.20 mm

### Data collection

**Table:**

Enraf–Nonius CAD-4 diffractometer	θ_max_ = 25º
ω/2θ scans	*h* = 0→8
Absorption correction: none	*k* = 0→15
2247 measured reflections	*l* = −15→15
2079 independent reflections	1 standard reflections
1296 reflections with *I* > 2σ(*I*)	every 30 min
*R*_int_ = 0.016	intensity decay: none

### Refinement

**Table:**

Refinement on *F*	H-atom parameters constrained
*R*[*F*^2^ > 2σ(*F*^2^)] = 0.049	*w* = 1/[σ^2^(*F*) + 0.0004*F*^2^]
*wR*(*F*^2^) = 0.052	(Δ/σ)_max_ = 0.003
*S* = 1.27	Δρ_max_ = 0.28 e Å^−^^3^
2079 reflections	Δρ_min_ = −0.28 e Å^−^^3^
145 parameters	Extinction correction: none

### Fractional atomic coordinates and isotropic or equivalent isotropic displacement parameters (Å^2^)

**Table:**

	*x*	*y*	*z*	*U*_iso_*/*U*_eq_	
O1	0.5397 (2)	0.2837 (1)	0.1016 (1)	0.0557 (5)	
O2	0.6667 (2)	−0.0303 (1)	0.4412 (1)	0.0595 (5)	
C1	0.4341 (3)	0.2236 (2)	0.2535 (2)	0.0417 (6)	
C2	0.4327 (3)	0.2060 (2)	0.1324 (2)	0.0416 (6)	
C3	0.5169 (3)	0.1035 (2)	0.1158 (2)	0.0429 (6)	
C4	0.4404 (3)	0.0172 (2)	0.1708 (2)	0.0447 (6)	
C5	0.4266 (3)	0.0387 (2)	0.2886 (2)	0.0438 (6)	
C6	0.6110 (3)	0.0420 (2)	0.3790 (2)	0.0426 (6)	
C7	0.7296 (3)	0.1362 (2)	0.3922 (2)	0.0430 (6)	
C8	0.6271 (3)	0.2343 (2)	0.3406 (2)	0.0452 (6)	
C9	0.3273 (3)	0.1393 (2)	0.2908 (2)	0.0477 (6)	
C10	0.7322 (3)	0.1007 (2)	0.1397 (2)	0.0522 (6)	
C11	0.8518 (3)	0.0511 (2)	0.2448 (2)	0.0498 (6)	
C12	0.8989 (3)	0.1092 (2)	0.3527 (2)	0.0535 (6)	
C13	0.9221 (4)	−0.0409 (2)	0.2436 (2)	0.0705 (8)	
C14	0.2323 (4)	0.2130 (2)	0.0531 (2)	0.0609 (7)	
H1O1	0.4677	0.3486	0.0866	0.056	
HC1	0.3655	0.2883	0.2542	0.042	
HC3	0.4643	0.0900	0.0338	0.043	
H1C4	0.5236	−0.0429	0.1766	0.045	
H2C4	0.3124	0.0005	0.1208	0.045	
HC5	0.3507	−0.0165	0.3074	0.044	
HC7	0.7811	0.1489	0.4744	0.043	
H1C8	0.7085	0.2710	0.3041	0.045	
H2C8	0.6119	0.2764	0.4033	0.045	
H1C9	0.1969	0.1358	0.2387	0.048	
H2C9	0.3247	0.1534	0.3682	0.048	
H1C10	0.7751	0.1726	0.1414	0.052	
H2C10	0.7558	0.0645	0.0757	0.052	
H1C12	0.9612	0.1738	0.3428	0.054	
H2C12	0.9872	0.0673	0.4122	0.054	
H1C13	0.8958	−0.0787	0.1715	0.071	
H2C13	1.0014	−0.0724	0.3147	0.071	
H1C14	0.1544	0.1594	0.0728	0.061	
H2C14	0.1798	0.2812	0.0604	0.061	
H3C14	0.2321	0.2029	−0.0256	0.061	

### Atomic displacement parameters (Å^2^)

**Table:**

	*U*^11^	*U*^22^	*U*^33^	*U*^12^	*U*^13^	*U*^23^
O1	0.080 (1)	0.0411 (9)	0.0500 (9)	−0.0072 (8)	0.0264 (9)	0.0068 (7)
O2	0.071 (1)	0.050 (1)	0.051 (1)	0.0052 (9)	0.0098 (9)	0.0139 (8)
C1	0.047 (1)	0.037 (1)	0.041 (1)	0.005 (1)	0.014 (1)	0.000 (1)
C2	0.052 (2)	0.034 (1)	0.038 (1)	−0.003 (1)	0.013 (1)	0.001 (1)
C3	0.054 (1)	0.040 (1)	0.033 (1)	−0.004 (1)	0.010 (1)	−0.006 (1)
C4	0.049 (2)	0.038 (1)	0.041 (1)	−0.005 (1)	0.005 (1)	−0.003 (1)
C5	0.044 (1)	0.042 (1)	0.045 (1)	−0.005 (1)	0.014 (1)	0.007 (1)
C6	0.052 (2)	0.044 (1)	0.034 (1)	0.002 (1)	0.018 (1)	0.002 (1)
C7	0.048 (1)	0.046 (1)	0.031 (1)	−0.002 (1)	0.008 (1)	−0.005 (1)
C8	0.059 (2)	0.038 (1)	0.038 (1)	−0.003 (1)	0.014 (1)	−0.006 (1)
C9	0.048 (1)	0.052 (1)	0.045 (1)	0.001 (1)	0.018 (1)	0.004 (1)
C10	0.062 (2)	0.055 (2)	0.045 (1)	−0.002 (1)	0.024 (1)	−0.007 (1)
C11	0.041 (1)	0.051 (1)	0.059 (2)	−0.003 (1)	0.019 (1)	−0.006 (1)
C12	0.045 (1)	0.059 (2)	0.052 (1)	−0.002 (1)	0.008 (1)	−0.008 (1)
C13	0.059 (2)	0.060 (2)	0.090 (2)	0.005 (1)	0.019 (2)	−0.008 (2)
C14	0.064 (2)	0.058 (2)	0.050 (1)	0.004 (1)	0.002 (1)	0.008 (1)

### Geometric parameters (Å, °)

**Table:**

O1—C2	1.432 (2)	C7—C8	1.544 (3)
O1—H1O1	1.000	C7—C12	1.551 (3)
O2—C6	1.223 (2)	C7—HC7	1.000
C1—C2	1.540 (3)	C8—H1C8	1.000
C1—C8	1.534 (3)	C8—H2C8	1.000
C1—C9	1.531 (3)	C9—H1C9	1.000
C1—HC1	1.000	C9—H2C9	1.000
C2—C3	1.536 (3)	C10—C11	1.501 (3)
C2—C14	1.535 (3)	C10—H1C10	1.000
C3—C4	1.536 (3)	C10—H2C10	1.000
C3—C10	1.560 (3)	C11—C12	1.503 (3)
C3—HC3	1.000	C11—C13	1.327 (3)
C4—C5	1.546 (3)	C12—H1C12	1.000
C4—H1C4	1.000	C12—H2C12	1.000
C4—H2C4	1.000	C13—H1C13	1.000
C5—C6	1.500 (3)	C13—H2C13	1.000
C5—C9	1.529 (3)	C14—H1C14	1.000
C5—HC5	1.000	C14—H2C14	1.000
C6—C7	1.511 (3)	C14—H3C14	1.000
			
C2—O1—H1O1	110.1	C8—C7—HC7	106.1
C2—C1—C8	115.7 (2)	C12—C7—HC7	106.1
C2—C1—C9	110.7 (2)	C1—C8—C7	117.6 (2)
C2—C1—HC1	107.1	C1—C8—H1C8	107.4
C8—C1—C9	108.7 (2)	C1—C8—H2C8	107.4
C8—C1—HC1	107.1	C7—C8—H1C8	107.4
C9—C1—HC1	107.1	C7—C8—H2C8	107.4
O1—C2—C1	109.3 (2)	H1C8—C8—H2C8	109.5
O1—C2—C3	107.5 (2)	C1—C9—C5	108.5 (2)
O1—C2—C14	107.8 (2)	C1—C9—H1C9	109.7
C1—C2—C3	113.1 (2)	C1—C9—H2C9	109.7
C1—C2—C14	109.6 (2)	C5—C9—H1C9	109.7
C3—C2—C14	109.3 (2)	C5—C9—H2C9	109.7
C2—C3—C4	111.2 (2)	H1C9—C9—H2C9	109.5
C2—C3—C10	116.5 (2)	C3—C10—C11	119.0 (2)
C2—C3—HC3	104.4	C3—C10—H1C10	107.0
C4—C3—C10	114.3 (2)	C3—C10—H2C10	107.0
C4—C3—HC3	104.4	C11—C10—H1C10	107.0
C10—C3—HC3	104.4	C11—C10—H2C10	107.0
C3—C4—C5	116.3 (2)	H1C10—C10—H2C10	109.5
C3—C4—H1C4	107.7	C10—C11—C12	118.8 (2)
C3—C4—H2C4	107.7	C10—C11—C13	121.5 (2)
C5—C4—H1C4	107.7	C12—C11—C13	119.6 (2)
C5—C4—H2C4	107.7	C7—C12—C11	114.9 (2)
H1C4—C4—H2C4	109.5	C7—C12—H1C12	108.1
C4—C5—C6	114.2 (2)	C7—C12—H2C12	108.1
C4—C5—C9	110.9 (2)	C11—C12—H1C12	108.1
C4—C5—HC5	107.9	C11—C12—H2C12	108.1
C6—C5—C9	107.8 (2)	H1C12—C12—H2C12	109.5
C6—C5—HC5	107.9	C11—C13—H1C13	120.0
C9—C5—HC5	107.9	C11—C13—H2C13	120.0
O2—C6—C5	121.1 (2)	H1C13—C13—H2C13	120.0
O2—C6—C7	119.9 (2)	C2—C14—H1C14	109.5
C5—C6—C7	119.1 (2)	C2—C14—H2C14	109.5
C6—C7—C8	116.2 (2)	C2—C14—H3C14	109.5
C6—C7—C12	107.2 (2)	H1C14—C14—H2C14	109.5
C6—C7—HC7	106.1	H1C14—C14—H3C14	109.5
C8—C7—C12	114.4 (2)	H2C14—C14—H3C14	109.5
			
H1O1—O1—C2—C1	−75.5	HC3—C3—C10—H2C10	−18.6
H1O1—O1—C2—C3	161.4	C3—C4—C5—C6	−72.0 (2)
H1O1—O1—C2—C14	43.6	C3—C4—C5—C9	50.0 (2)
C8—C1—C2—O1	−53.4 (2)	C3—C4—C5—HC5	168.0
C8—C1—C2—C3	66.3 (2)	H1C4—C4—C5—C6	49.0
C8—C1—C2—C14	−171.4 (2)	H1C4—C4—C5—C9	171.0
C9—C1—C2—O1	−177.7 (2)	H1C4—C4—C5—HC5	−71.0
C9—C1—C2—C3	−58.0 (2)	H2C4—C4—C5—C6	167.0
C9—C1—C2—C14	64.3 (2)	H2C4—C4—C5—C9	−71.0
HC1—C1—C2—O1	65.9	H2C4—C4—C5—HC5	47.0
HC1—C1—C2—C3	−174.4	C4—C5—C6—O2	−100.9 (2)
HC1—C1—C2—C14	−52.1	C4—C5—C6—C7	78.6 (2)
C2—C1—C8—C7	−85.4 (2)	C9—C5—C6—O2	135.4 (2)
C2—C1—C8—H1C8	35.7	C9—C5—C6—C7	−45.1 (2)
C2—C1—C8—H2C8	153.4	HC5—C5—C6—O2	19.1
C9—C1—C8—C7	39.9 (2)	HC5—C5—C6—C7	−161.4
C9—C1—C8—H1C8	161.0	C4—C5—C9—C1	−57.7 (2)
C9—C1—C8—H2C8	−81.3	C4—C5—C9—H1C9	62.2
HC1—C1—C8—C7	155.3	C4—C5—C9—H2C9	−177.5
HC1—C1—C8—H1C8	−83.6	C6—C5—C9—C1	68.0 (2)
HC1—C1—C8—H2C8	34.1	C6—C5—C9—H1C9	−172.2
C2—C1—C9—C5	62.6 (2)	C6—C5—C9—H2C9	−51.9
C2—C1—C9—H1C9	−57.2	HC5—C5—C9—C1	−175.7
C2—C1—C9—H2C9	−177.5	HC5—C5—C9—H1C9	−55.8
C8—C1—C9—C5	−65.6 (2)	HC5—C5—C9—H2C9	64.5
C8—C1—C9—H1C9	174.6	O2—C6—C7—C8	−160.0 (2)
C8—C1—C9—H2C9	54.3	O2—C6—C7—C12	70.7 (2)
HC1—C1—C9—C5	179.0	O2—C6—C7—HC7	−42.4
HC1—C1—C9—H1C9	59.2	C5—C6—C7—C8	20.5 (3)
HC1—C1—C9—H2C9	−61.1	C5—C6—C7—C12	−108.8 (2)
O1—C2—C3—C4	167.3 (2)	C5—C6—C7—HC7	138.1
O1—C2—C3—C10	34.0 (2)	C6—C7—C8—C1	−17.6 (3)
O1—C2—C3—HC3	−80.6	C6—C7—C8—H1C8	−138.8
C1—C2—C3—C4	46.6 (2)	C6—C7—C8—H2C8	103.6
C1—C2—C3—C10	−86.8 (2)	C12—C7—C8—C1	108.2 (2)
C1—C2—C3—HC3	158.7	C12—C7—C8—H1C8	−13.0
C14—C2—C3—C4	−75.8 (2)	C12—C7—C8—H2C8	−130.7
C14—C2—C3—C10	150.8 (2)	HC7—C7—C8—C1	−135.2
C14—C2—C3—HC3	36.2	HC7—C7—C8—H1C8	103.6
O1—C2—C14—H1C14	180.0	HC7—C7—C8—H2C8	−14.0
O1—C2—C14—H2C14	−60.0	C6—C7—C12—C11	45.4 (2)
O1—C2—C14—H3C14	60.0	C6—C7—C12—H1C12	166.2
C1—C2—C14—H1C14	−61.1	C6—C7—C12—H2C12	−75.4
C1—C2—C14—H2C14	58.9	C8—C7—C12—C11	−84.9 (2)
C1—C2—C14—H3C14	178.9	C8—C7—C12—H1C12	35.9
C3—C2—C14—H1C14	63.4	C8—C7—C12—H2C12	154.3
C3—C2—C14—H2C14	−176.6	HC7—C7—C12—C11	158.4
C3—C2—C14—H3C14	−56.6	HC7—C7—C12—H1C12	−80.8
C2—C3—C4—C5	−43.4 (2)	HC7—C7—C12—H2C12	37.6
C2—C3—C4—H1C4	−164.4	C3—C10—C11—C12	−80.0 (3)
C2—C3—C4—H2C4	77.6	C3—C10—C11—C13	102.6 (3)
C10—C3—C4—C5	91.0 (2)	H1C10—C10—C11—C12	41.4
C10—C3—C4—H1C4	−30.0	H1C10—C10—C11—C13	−136.0
C10—C3—C4—H2C4	−148.0	H2C10—C10—C11—C12	158.7
HC3—C3—C4—C5	−155.5	H2C10—C10—C11—C13	−18.7
HC3—C3—C4—H1C4	83.5	C10—C11—C12—C7	63.8 (3)
HC3—C3—C4—H2C4	−34.5	C10—C11—C12—H1C12	−57.0
C2—C3—C10—C11	105.5 (2)	C10—C11—C12—H2C12	−175.4
C2—C3—C10—H1C10	−15.8	C13—C11—C12—C7	−118.7 (2)
C2—C3—C10—H2C10	−133.1	C13—C11—C12—H1C12	120.5
C4—C3—C10—C11	−26.4 (3)	C13—C11—C12—H2C12	2.1
C4—C3—C10—H1C10	−147.8	C10—C11—C13—H1C13	0.0
C4—C3—C10—H2C10	94.9	C10—C11—C13—H2C13	−180.0
HC3—C3—C10—C11	−139.9	C12—C11—C13—H1C13	−177.4
HC3—C3—C10—H1C10	98.7	C12—C11—C13—H2C13	2.6

### Hydrogen-bond geometry (Å, °)

**Table:**

*D*—H···*A*	*D*—H	H···*A*	*D*···*A*	*D*—H···*A*
O1—H1O1···O2^i^	1.00	1.87	2.867 (4)	180

Symmetry codes: (i) −*x*+1, *y*+1/2, −*z*+1/2.
